# Artificial Intelligence–Enabled ECG Screening for LVSD in LBBB: Evaluating Model Development and Transfer Learning Approaches

**DOI:** 10.1016/j.jacadv.2025.102089

**Published:** 2025-08-21

**Authors:** Hak Seung Lee, Sooyeon Lee, Sora Kang, Ga In Han, Ah-Hyun Yoo, Jong-Hwan Jang, Yong-Yeon Jo, Jeong Min Son, Min Sung Lee, Joon-myoung Kwon, Kyung-Hee Kim

**Affiliations:** a Digital Healthcare Institute, Sejong Medical Research Institute, Bucheon, Republic of Korea; b Medical AI Co, Ltd, Seoul, Republic of Korea; c Division of Cardiology, Department of Internal Medicine, Incheon Sejong Hospital, Cardiovascular Center, Incheon, Republic of Korea

**Keywords:** artificial intelligence, electrocardiogram, left bundle branch block, left ventricular systolic dysfunction

## Abstract

**Background:**

Left bundle branch block (LBBB) is a common electrocardiogram (ECG) abnormality associated with left ventricular systolic dysfunction (LVSD). Although artificial intelligence (AI)–driven ECG analysis shows promise for LVSD screening, it remains unclear if a general AI-ECG model or one tailored for LBBB patients yields better performance.

**Objectives:**

This study evaluates 4 AI-ECG models for detecting LVSD in LBBB patients and examines the impact of training cohort definitions.

**Methods:**

We developed 4 models using 364,845 ECGs from 4 hospitals: 1) a general AI-ECG model; 2) a model trained on automatically extracted LBBB cases; 3) a model trained on a well-curated single-center LBBB data set with expert review; and 4) a hybrid model employing transfer learning by fine-tuning the general model with single-center LBBB data. LVSD was defined as an ejection fraction ≤40%. All models were externally validated on 1,334 ECGs from another hospital, with performance assessed by area under the receiver operating characteristic curve (AUROC), sensitivity, specificity, and predictive values.

**Results:**

In external validation, the transfer learning model achieved the highest AUROC (0.903; 95% CI: 0.887-0.918), closely followed by the general model (0.899; 95% CI: 0.883-0.915); the difference was not significant. Models using automated or expert-based LBBB extraction had lower AUROCs (0.879 and 0.841, respectively). The general model demonstrated high sensitivity, whereas the transfer learning model exhibited superior specificity.

**Conclusions:**

Our findings indicate that a broad AI-ECG model reliably detects LVSD in LBBB patients, and transfer learning offers modest improvements without requiring curated LBBB data sets. Evaluating algorithms in representative clinical populations is essential.

Left bundle branch block (LBBB) is an electrocardiographic abnormality with a prevalence ranging from approximately 0.1% to 1.0% in the general population, increasing with age.^1^^,^^2^ LBBB leads to abnormal ventricular activation and contraction, resulting in left ventricular (LV) dyssynchrony, which is considered a key contributor to LV systolic dysfunction (LVSD).^3^, ^4^, ^5^ However, not all patients with LBBB exhibit impaired cardiac function, and studies suggest that the prevalence of LV dysfunction in LBBB patients with previously preserved ejection fraction (EF) ranges from 17% to 38% over long-term follow-up.^6^^,^^7^ These findings underscore the complex interplay between LBBB and LV dysfunction, highlighting the need for early and accurate LVSD detection in this high-risk group.

With advancements in artificial intelligence–enabled electrocardiogram (AI-ECG) analysis, these technologies have been reported to enable precise phenotyping and characterization of subtle cardiac physiological changes.^8^ A representative application is deep learning models for LVSD screening, and previous studies have primarily focused on training models using large, heterogeneous data sets.^9^, ^10^, ^11^, ^12^ While this approach enhances generalizability, the unique ECG patterns and distinct clinical profiles of LBBB patients raise concerns that models trained on broad, heterogeneous data sets may not achieve optimal accuracy when applied to this subgroup.^13^ Therefore, investigating how subtle variations in case definitions and cohort selection impact each model's performance is crucial.^14^, ^15^, ^16^

The objective of this study was to determine whether an AI-ECG model tailored specifically for patients with LBBB can achieve superior diagnostic accuracy for detecting LVSD compared to a general AI-ECG model. To address this, we developed and evaluated multiple models—including a general model, a dedicated LBBB-specific model, and a hybrid model using transfer learning—while keeping the underlying architecture and input data constant. This approach enabled us to isolate the impact of training data set composition and specialized model development on the model's performance in LBBB patients.

## Methods

### Data source and study population

**Figure 1:**
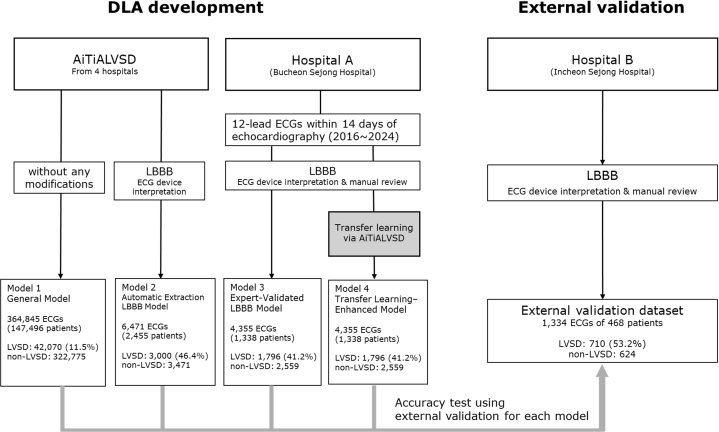
Study Flow Model 1 (General model) and Model 2 (Automatic Extraction LBBB model) originated from a large multihospital data set, whereas Model 3 (Expert-Validated LBBB model) and Model 4 (Transfer Learning-Enhanced model) were developed using a well-curated LBBB data set. Model 4 is a fine-tuned model that leverages transfer learning by using the pretrained weights from the general Model 1. ECG = electrocardiogram; LBBB = left bundle branch block; LVSD = left ventricular systolic dysfunction.

This study evaluated the accuracy of 4 different AI-ECG models for detecting LVSD by validating them on an external cohort (Figure 1). One model had been previously developed, while 3 additional models were trained using 12-lead ECGs from the original model's development set and 2 hospitals: hospital A (Bucheon Sejong Hospital) and hospital B (Incheon Sejong Hospital). For hospital A, we included all patients who underwent an echocardiogram and had a corresponding ECG within 14 days between January 2016 and December 2024. Similarly, we extracted all 12-lead ECGs meeting the same criteria for hospital B between January 2017 and December 2024. All ECGs were recorded at a 500 Hz sampling rate, with a standard 10-second, 12-lead configuration, and were stored in the MUSE Cardiology Information System (GE Healthcare). The PAGEWRITER TC30 and TC70 devices (Philips) were used for ECG acquisition, and electrocardiographic features were extracted accordingly.

All procedures involving human participants were performed in accordance with the ethical standards of Bucheon Sejong Hospital and Incheon Sejong Hospital's Institutional Review Boards (BSH 2024-09-003-005 and ISH 2024-09-001), as well as with the Helsinki Declaration and its later amendments. Both Institutional Review Boards waived the requirement for written informed consent due to the retrospective nature of the study, the fully anonymized data set, and minimal risk to patients.

### ECG labeling and LBBB definition

To evaluate how the choice of development set influences model performance, we compared multiple models developed using different cohorts. The training data sets varied in how the LBBB cases were identified—ranging from automated extraction to expert-validated selection. The broadest LBBB definition was based on automated ECG interpretation from the MUSE Cardiology Information System, which identified LBBB-positive ECGs through its automated screening process. From a total of 364,845 ECGs, 6,547 cases (1.79%) were flagged as LBBB. For both hospitals—one serving as the development set and the other as the external validation set—LBBB status was initially determined using the automated screening from the MUSE Cardiology Information System. In some cases, this automated labeling alone was used for model development. In others, additional manual review by board-certified technicians and cardiologists was applied to confirm or exclude LBBB morphology. This expert review ensured accurate identification of LBBB cases. The use of automated-only vs expert-confirmed labeling strategies was later compared across models, and their influence on model performance was quantitatively evaluated.

LVSD was defined based on the LV EF measured from the final echocardiography report, which employed the modified Simpson's method. A threshold of EF ≤40% was used to indicate LVSD.^17^^,^^18^ If multiple echocardiograms were performed within 14 days of the ECG, the one closest in time to the ECG was selected as the index study.

### AI-ECG model design and training

All 4 models output a continuous LVSD probability score (0-100) based on the final activation layer. For binary classification, a fixed cutoff was applied, determined by the Youden Index on a validation set. To improve the reliability of the probability estimates, temperature scaling was used for score calibration. Additional details on the calibration method, score distribution (visualized in Supplemental Figure 1), and threshold selection are provided in Supplemental Methods 1.

In our previous study, we developed and validated an AI-ECG model for LVSD detection, referred to as AiTiALVSD, across multiple environments.^11^^,^^19^^,^^20^ Model 1 (AiTiALVSD) was built to predict LVSD using only digital signals extracted from 12-lead ECGs. It was developed using a residual neural network architecture and trained on 364,845 ECGs collected from 4 hospitals in the Republic of Korea without incorporating additional clinical variables.^19^ The model architecture was based on a ResNet variant tailored for 12-lead ECG input. Each ECG was represented as a 12 × 5,000 matrix (12 leads, 10 seconds at 500 Hz). The network consisted of 3 convolutional stages, each comprising 4 residual blocks with batch normalization, ReLU activation, and skip connections. A global average pooling layer was followed by a fully connected layer and a final sigmoid activation to produce a probability score for LVSD classification. This artificial intelligence/machine learning (AI/ML)-based software-as-a-medical-device has received approval from the Ministry of Food and Drug Safety of the Republic of Korea and has been designated an “Innovative Medical Device,” analogous to the Breakthrough Device Program in the United States. The model outputs an LVSD probability score ranging from 0 to 100, with a precision of one decimal place, allowing for a continuous risk assessment. The architecture of the AI-ECG model has been described in detail in previous publications.^19^^,^^20^

To explore LBBB-specific performance, we expanded the model framework by incorporating 3 additional models. Model 2 was developed using 6,471 ECGs (1.77% of the total 364,845) from the original AiTiALVSD development set that were identified as LBBB-positive based solely on the presence of the interpretation phrase “LBBB” or “Left bundle branch block” in the MUSE-generated automated report. This model was designed to assess whether training exclusively on preidentified LBBB cases from a large, heterogeneous data set could improve LVSD detection in LBBB patients. Model 3 was an LBBB-specific model trained using 6,558 ECGs from hospital A, where LBBB status was confirmed through both automated ECG interpretation and manual expert review; within this data set, 1,796 ECGs were labeled as LVSD and 2,559 as non-LVSD. This model was built using the same neural network architecture as Model 1 to ensure direct comparability.

Model 4 employed a transfer learning approach in which the AiTiALVSD (Model 1) weights served as a pretrained starting point, and the model was then fine-tuned on the LBBB data set from hospital A. Given the relative scarcity of well-annotated LBBB cases within the broader heart failure population, assembling large training data sets is challenging. Transfer learning leverages knowledge acquired from large, diverse data sets to improve performance on related tasks with limited data, making it a promising solution for adapting AI models to small, specialized cohorts.^21^^,^^22^ Building on our prior work developing and validating an AI-ECG model for LVSD detection across multiple subgroups, we fine-tuned Model 1 on the LBBB data set from hospital A, thereby adapting the general model to the specific ECG characteristics of LBBB patients. This strategy aimed to adapt the general model to a specialized cohort, improving performance in LBBB patients while leveraging the broader learned features from Model 1. By evaluating these 4 models, this study aimed to determine the most effective strategy for LVSD detection in LBBB patients, examining whether a generalizable AI-ECG model, a dedicated LBBB-specific model, or a transfer learning adaptation yields the highest diagnostic accuracy. Full details of the fine-tuning protocol, including training hyperparameters and layer update strategies, are provided in Supplemental Methods 2.

### Evaluation and external validation

The comparative analysis aimed to determine the most effective strategy for accurate LVSD detection in LBBB patients across different modeling approaches. An external validation set from hospital B, consisting of 1,334 ECGs (710 with LVSD and 624 without LVSD), was reserved to assess the final model performance. All 4 models—the general AI-ECG model (AiTiALVSD, Model 1), the automatic extraction LBBB model (Model 2), the expert-validated LBBB model (Model 3), and the transfer learning-enhanced LBBB model (Model 4)—were evaluated on this held-out cohort to measure their diagnostic accuracy in predicting LVSD. Performance metrics included the area under the receiver operating characteristic curve (AUROC) and the area under the precision-recall curve, with 95% CIs, as well as sensitivity, specificity, positive predictive value (PPV), and negative predictive value (NPV) at various operating thresholds.

Additionally, to evaluate model performance at a clinically relevant threshold, we fixed sensitivity at 90% for each model. For each, we determined the probability-score cutoff on the held-out test set that yielded 0.90 sensitivity, then calculated the corresponding specificity.^23^ All analyses used the same cohort and procedures as our AUROC calculations.

### Explainable AI

To better understand how the AI-ECG model detects LVSD in LBBB patients, we applied the Generative Counterfactual ECG (GCX) framework.^24^ This method generates modified ECG waveforms that change the model's prediction while preserving physiological plausibility. GCX produces a series of overlaid ECG traces colored from black (original) through increasingly intense reds, with each shade representing a minimal, physiologically constrained edit—such as subtle R-wave augmentation or S-wave narrowing—that incrementally raises the AI-ECG score. Specifically, to illustrate how the model interprets ECG signals when predicting LVSD, we applied GCX to a representative case: we selected a median beat from a 10-second ECG and iteratively modified the waveform in a physiologically plausible manner, progressively increasing the AI-ECG score. This process allowed us to visualize exactly how waveform features evolved to drive the model's classification of the ECG as LVSD, providing insight into its decision boundaries.

We performed exploratory univariate correlation analyses between conventional ECG parameters and AI-ECG scores to identify features potentially associated with AI-predicted LVSD. Eight ECG parameters—including heart rate, PR interval (time from the onset of the P wave to the start of the QRS complex), QRS duration, QT/QTc intervals, and P-, R-, and T-wave axes—were evaluated using Pearson correlation coefficients with two-tailed significance testing and Bonferroni correction. Multivariable adjustment was not performed, as the goal was hypothesis generation rather than inference.

### Statistical analysis

Continuous variables were reported as mean ± SD or median (IQR) as appropriate, and categorical variables as counts (%). Model performance was primarily evaluated using the AUROC. Statistical significance between AUROCs was assessed using the DeLong test. We computed performance metrics as follows: sensitivity was defined as the ratio of true positives to the sum of true positives and false negatives; specificity as true negatives divided by the sum of true negatives and false positives; PPV as true positives divided by the sum of true positives and false positives; and NPV as true negatives divided by the sum of true negatives and false negatives. In addition, to evaluate the predictive role of the AI-ECG, we assessed future LVSD events in both the false positive and true negative groups predicted by Model 1 (general model, AiTiALVSD) using Kaplan-Meier survival curves. For this analysis, all follow-up echocardiography data from the LBBB patient cohort were collected. We defined the time-to-event as the number of days between the index ECG and the first follow-up echocardiogram showing an LVEF ≤40%. If multiple echocardiograms were available, the earliest qualifying one was used. Patients who did not meet this criterion during follow-up were censored at the date of their last available echocardiogram. To complement the Kaplan-Meier survival analysis, we also conducted a multivariable Cox proportional hazards regression to estimate the adjusted HR for incident LVSD. The binary AI-ECG result (positive vs negative) was included as the primary predictor, along with clinical covariates: age, sex, diabetes mellitus (DM), hypertension, ischemic heart disease (IHD), heart failure, atrial fibrillation, chronic kidney disease, and stroke. The proportional hazards assumption was tested and satisfied for all covariates.

Moreover, several subgroup analyses were conducted to evaluate model performance across clinically meaningful strata, including age, sex, hypertension, DM, IHD, prior heart failure, and atrial fibrillation. Subgroup AUROC values were compared using the DeLong test, and the corresponding *P* values were used to assess potential differences in model performance across clinical strata. Finally, sensitivity maps were generated to highlight key features influencing the AI-ECG model's decisions. All statistical analyses were performed in Python (version 3.7.0) and R (version 4.2.2).

## Results

### Baseline characteristics of the external validation group

The external validation cohort consisted of 1,334 ECGs from 468 patients, with 710 (53.2%) classified as LVSD and 624 (46.8%) as non-LVSD (Table 1). Patients with LVSD had a mean EF of 25.7%, whereas the non-LVSD group had a mean EF of 57.2%. In terms of clinical characteristics, LVSD patients were more likely to be male, and had a higher prevalence of DM, prior heart failure, atrial fibrillation, and chronic kidney disease compared to the non-LVSD group. There was no significant difference in the prevalence of IHD between the 2 groups. Electrocardiographically, LVSD patients exhibited a significantly higher heart rate (78.6 beats/min vs 72.1 beats/min) and prolonged QRS duration (164.7 ms vs 148.9 ms). Additionally, QTc interval was longer in the LVSD group (513.7 ms vs 491.4 ms), reflecting greater electrical instability.

**Table 1:** Baseline Characteristics of External Validation Set According to LVSD

	LBBB			*P* Value
	Total (N = 1,334)	LVSD (n = 710)	Non-LVSD (n = 624)	
Age, y	70.9 ± 13.3	71.4 ± 12.3	70.9 ± 13.3	0.502
Male	571 (42.8)	354 (49.9)	217 (34.8)	<0.001
Height	157.7 ± 11.7	160.5 ± 10.1	157.7 ± 11.7	<0.001
Weight	61.1 ± 11.9	61.4 ± 13.5	61.1 ± 11.9	0.624
BMI, kg/m^2^	24.8 ± 9.3	23.9 ± 6.6	24.8 ± 9.3	0.033
Past medical illness				
Diabetes mellitus	357 (26.8)	220 (31.0)	137 (22.0)	0.008
Hypertension	644 (48.3)	329 (46.3)	315 (50.5)	0.684
Ischemic heart disease	627 (47.0)	342 (48.2)	285 (45.7)	0.068
Heart failure	913 (68.4)	573 (80.7)	340 (54.5)	<0.001
Atrial fibrillation	323 (24.2)	217 (30.6)	106 (17.0)	<0.001
Chronic kidney disease	164 (12.3)	118 (16.6)	46 (7.4)	<0.001
Stroke	141 (10.6)	89 (12.5)	52 (8.3)	0.184
Electrocardiogram				
HR, beats/min	72.1 ± 17.7	78.6 ± 19.1	72.1 ± 17.7	<0.001
PR interval, ms	188.5 ± 47.0	185.7 ± 45.2	188.5 ± 47.0	0.323
QT interval, ms	456.7 ± 52.5	457.0 ± 53.3	456.7 ± 52.5	0.934
QRS duration, ms	148.9 ± 15.5	164.7 ± 24.9	148.9 ± 15.5	<0.001
QTc interval, ms	491.4 ± 35.6	513.7 ± 40.3	491.4 ± 35.6	<0.001
P axis	43.9 ± 40.7	54.0 ± 49.6	43.9 ± 40.7	<0.001
R axis	−4.2 ± 33.2	−9.1 ± 34.4	−4.2 ± 33.2	0.009
T axis	111.8 ± 56.1	138.3 ± 66.5	118.1 ± 56.1	<0.001
LVEF	57.2 ± 8.7	25.7 ± 6.8	57.2 ± 8.7	<0.001
AiTiALVSD score	42.5 ± 32.2	63.2 ± 27.1	19.0 ± 18.4	<0.001

Values are n (%) or mean ± SD. The AiTiALVSD score (model 1, general model) outputs an LVSD probability score ranging from 0 to 100, with a precision of one decimal place.

BMI = body mass index; HR = heart rate; LBBB = left bundle branch block; LVEF = left ventricular ejection fraction; LVSD = left ventricular systolic dysfunction.

Baseline characteristics of the development set are summarized in Supplemental Table 1. Overall, the external validation cohort showed comparable distributions in demographics, past medical history, and ECG parameters to those in the development data set.

### Model performance in LVSD detection

All 4 AI-ECG models demonstrated high performance in detecting LVSD among LBBB patients. The transfer learning–enhanced model (Model 4) achieved the highest AUROC of 0.903 (95% CI: 0.887-0.918), followed by the general model (Model 1) at 0.899 (95% CI: 0.883-0.915). However, this difference between the 2 models was not statistically significant. The automatic extraction LBBB model (Model 2) and the expert-validated LBBB model (Model 3) had lower AUROC values of 0.879 (95% CI: 0.862-0.896) and 0.841 (95% CI: 0.820-0.903), respectively (Central Illustration, Table 2). Pairwise AUROC comparisons using the DeLong test confirmed that the difference between Models 1 and 4 was not statistically significant (*P* = 0.43), while comparisons between Models 1 and 2 (*P* = 0.003) and Models 1 and 3 (*P* < 0.001) reached statistical significance.

**Central Illustration:**
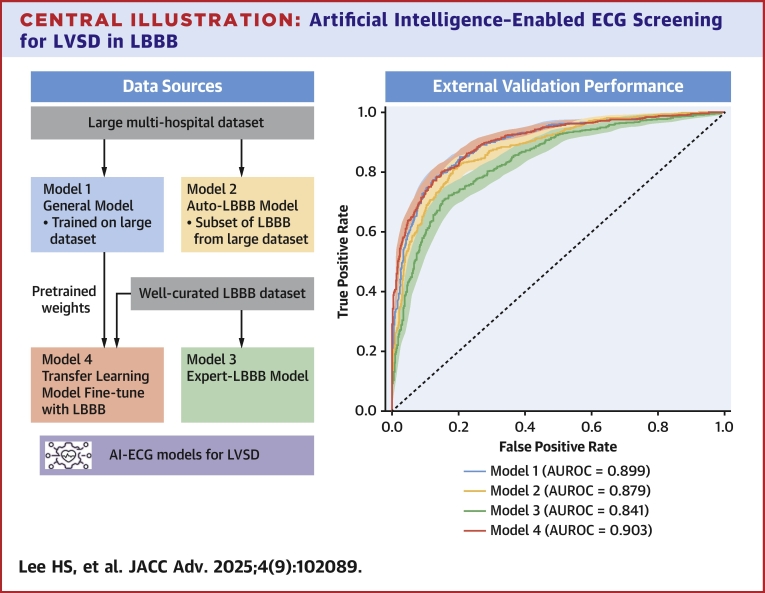
Artificial Intelligence–Enabled ECG Screening for LVSD in LBBB This figure presents the AUROC comparing the diagnostic performance of the 4 AI-ECG models in detecting LVSD among a well-curated external validation LBBB patient set. The curves illustrate the area under the ROC (AUROC) along with 95% CIs for each model: Model 1 (General Model), Model 2 (Automatic Extraction LBBB Model), Model 3 (Expert-Validated LBBB Model), and Model 4 (Transfer Learning–Enhanced Model). Abbreviations as in Figures 1 and 3.

**Table 2:** Performance Metrics of AI-ECG Models

	AUROC (95% CI)	AUPRC (95% CI)	Sensitivity (95% CI)	Specificity (95% CI)	PPV (95% CI)	NPV (95% CI)	Accuracy (95% CI)
Model 1	0.899 (0.883-0.915)	0.914 (0.896-0.930)	0.966 (0.950-0.978)	0.413 (0.377-0.452)	0.651 (0.623-0.679)	0.914 (0.878-0.944)	0.707 (0.684-0.731)
Model 2	0.879 (0.862-0.896)	0.894 (0.874-0.913)	0.831 (0.803-0.858)	0.785 (0.752-0.819)	0.814 (0.785-0.843)	0.803 (0.771-0.833)	0.809 (0.787-0.831)
Model 3	0.841 (0.820-0.863)	0.859 (0.832-0.884)	0.722 (0.688-0.756)	0.826 (0.797-0.856)	0.825 (0.793-0.854)	0.724 (0.690-0.758)	0.771 (0.743-0.795)
Model 4	0.903 (0.887-0.918)	0.923 (0.908-0.937)	0.72 (0.687-0.753)	0.903 (0.881-0.928)	0.894 (0.868-0.919)	0.739 (0.708-0.772)	0.806 (0.786-0.827)

AI-ECG = artificial intelligence–enabled electrocardiogram; AUPRC = area under the precision-recall curve; AUROC = area under the receiver operating characteristic curve; NPV = negative predictive value; PPV = positive predictive value.

Model 1 had the highest sensitivity (0.966; 95% CI: 0.950-0.978) and the lowest specificity (0.413; 95% CI: 0.377-0.452). In contrast, Model 4 had higher specificity (0.903; 95% CI: 0.881-0.928) but lower sensitivity (0.720; 95% CI: 0.687-0.753). Models 2 and 3 demonstrated intermediate sensitivity and specificity values, positioned between the high-sensitivity/low-specificity profile of Model 1 and the low-sensitivity/high-specificity profile of Model 4. Analysis of predictive values showed that Model 4 had the highest PPV at 0.894 (95% CI: 0.868-0.919), whereas Model 1 had the highest NPV at 0.914 (95% CI: 0.878-0.944). Model 2 and Model 3 had intermediate PPV and NPV values. The specificity at 90% sensitivity for each model is summarized in Table 3. Models 1 and 4 exhibited greater specificity than Models 2 and 3, with Model 4 reaching 0.718 (probability score cutoff 29.3) and Model 1 reaching 0.702 (cutoff 23.5). In comparison, Models 2 and 3 yielded lower specificity values of 0.596 (cutoff 19.2) and 0.542 (cutoff 1.1), respectively.

**Table 3:** Specificity at Fixed 90% Sensitivity for LVSD Detection in LBBB Patients

	AUROC	Accuracy	Sensitivity	Specificity	Probability-Score Cutoff (at Sensitivity = 90%)
Model 1	0.899	0.807	0.90	0.702	23.5
Model 2	0.879	0.758	0.90	0.596	19.2
Model 3	0.841	0.732	0.90	0.542	1.1
Model 4	0.903	0.815	0.90	0.718	29.3

Abbreviations as in Tables 1 and 2.

### Predictive role of AI-ECG in future LVSD events

**Figure 2:**
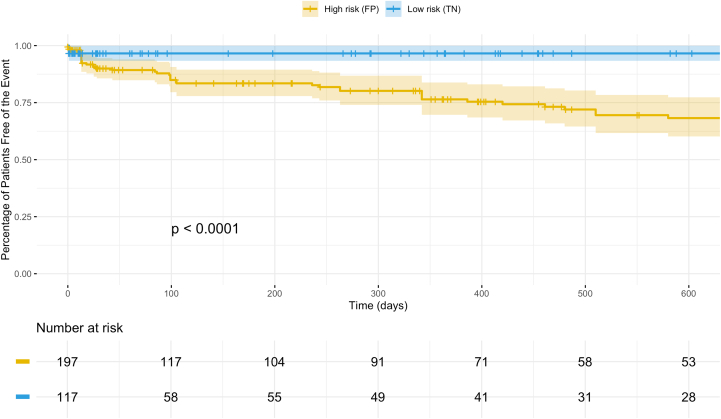
Kaplan-Meier Survival Analysis for Future LVSD Events This figure displays Kaplan-Meier curves demonstrating the cumulative incidence of future LVSD events during follow-up among LBBB patients, stratified by predictions of Model 1 (general model, AiTiALVSD). The analysis shows that those with false positive predictions had a significantly higher incidence of subsequent LVSD (38%) compared to those with true negative results (8.5%; *P* < 0.0001). FP = false positive; TN = true negative; other abbreviations as in Figure 1.

To evaluate the predictive role of AI-ECG, we assessed future LVSD events in the false positive and true negative groups as classified by Model 1 (general model, AiTiALVSD) using Kaplan-Meier survival curves (Figure 2). The median follow-up duration was 117 days (IQR: 24-280 days). During follow-up, the cumulative incidence of LVSD reached 38% in the false positive group and 8.5% in the true negative group (*P* < 0.0001). These findings demonstrate that even among patients with initially preserved EF, a positive AI-ECG result is associated with a substantially increased long-term risk of developing LVSD, highlighting its utility for early risk stratification.

To further examine the independent prognostic value of the AI-ECG result, we conducted a multivariable Cox proportional hazards regression analysis (Table 4). After adjusting for age, sex, and comorbidities, a positive AI-ECG remained strongly associated with incident LVSD (HR: 5.16; 95% CI: 3.39-7.86; *P* < 0.001). Additional independent predictors included history of heart failure (HR: 1.51; *P* < 0.001) and chronic kidney disease (HR: 1.39; *P* = 0.008).

**Table 4:** Multivariable Cox Proportional Hazards Regression for Incident LVSD After Baseline ECG

	HR	95% CI	*P* Value
AI-ECG positive	5.16	3.39-7.86	<0.001
Age	1.00	1.00-1.01	0.278
Male	1.07	0.91-1.27	0.414
Diabetes mellitus	1.12	0.92-1.37	0.249
Hypertension	0.82	0.68-0.98	0.029
Ischemic heart disease	1.02	0.86-1.22	0.820
Heart failure	1.51	1.21-1.87	<0.001
Atrial fibrillation	1.08	0.89-1.30	0.441
Chronic kidney disease	1.39	1.09-1.78	0.008
Stroke	0.94	0.72-1.22	0.629

Abbreviations as in Tables 1 and 2.

### Subgroup analysis of the general model

**Figure 3:**
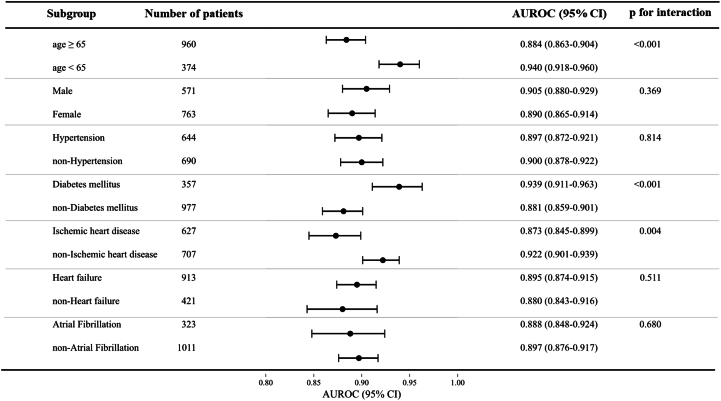
Subgroup Analysis of the General AI-ECG Model This forest plot summarizes the diagnostic performance (AUROC with 95% CI) of the general AI-ECG model across various patient subgroups. Overall, the model demonstrated consistently high AUROC values across all subgroups, indicating robust diagnostic performance. AI-ECG = artificial intelligence–enabled electrocardiogram; AUROC = area under the receiver operating characteristic curve.

Subgroup analysis was performed to assess the general model's performance across different patient characteristics (Figure 3, Supplemental Table 2). Overall, the model demonstrated consistently high AUROC values across all subgroups, indicating robust diagnostic performance. However, statistically significant differences were observed in 3 subgroups: age, DM, and IHD. The model showed higher AUROC in patients aged <65 years (0.940) compared to ≥65 years (0.884), in DM patients (0.939) compared to non-DM patients (0.881), and in non-IHD patients (0.922) compared to IHD patients (0.873).

### Explainable AI analysis

**Figure 4:**
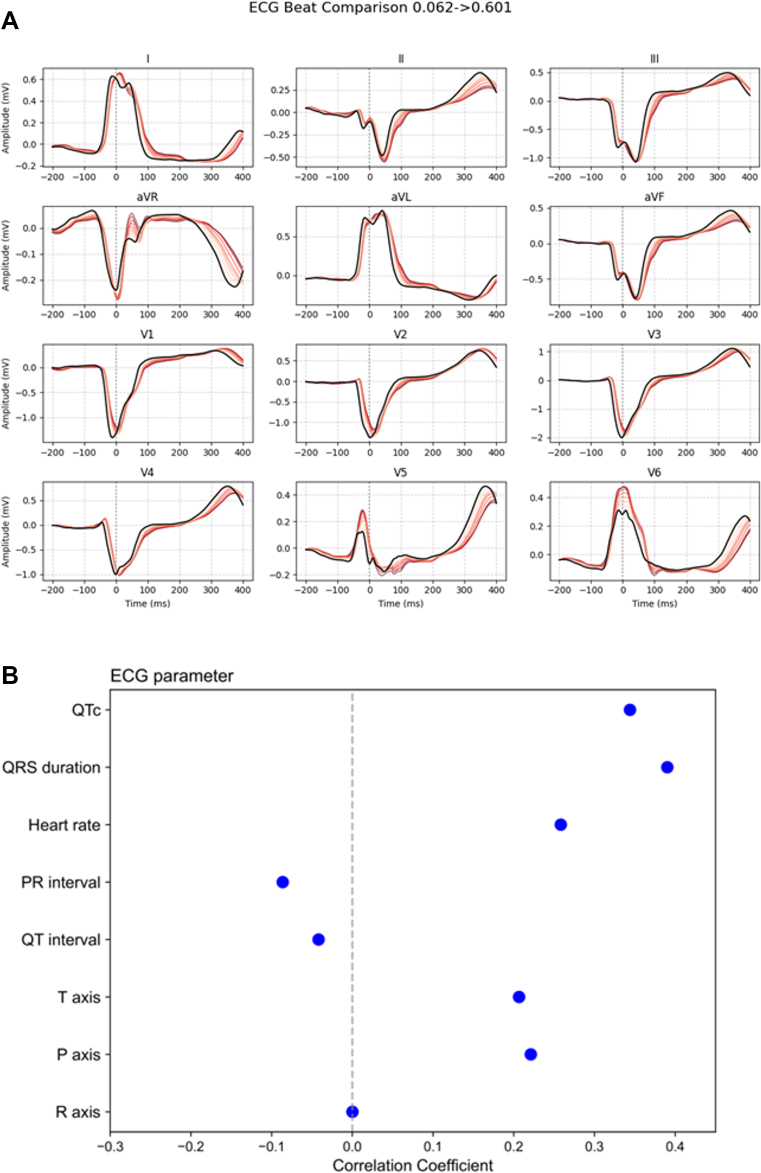
ECG Morphologies Associated With AI-ECG–Predicted LVSD in Patients With LBBB (A) The Generative Counterfactual ECG (GCX) framework was used to create modified ECGs that progressively increased the AI-ECG–predicted LVSD probability while preserving physiological plausibility. As the predicted score increased, characteristic changes such as prolonged QRS duration, QTc interval, and altered T-wave morphology were observed, indicating the model's reliance on these features for LVSD detection. (B) Univariate correlation analysis confirmed significant associations (*P* < 0.001) between AI-ECG scores and conventional ECG parameters, including QRS duration and QTc interval, reinforcing their importance in the model's decision-making process. GCX = Generative Counterfactual ECG Explainability Framework; ms = millisecond; other abbreviations as in Figures 1 and 3.

To illustrate how the model interprets ECG signals when predicting LVSD, we applied the GCX framework to a representative case (Figure 4A). The GCX-based waveform edits revealed that the model raises the LVSD probability primarily by augmenting R-wave amplitude and flattening the S-wave. In the limb leads (I, II, aVR, and aVL), the R-wave gradually became larger while the terminal S-wave grew shallower. In the precordial leads V_1_ through V_3_, the characteristically deep S-wave of LBBB narrowed and the R-wave amplitude increased. Finally, in the lateral leads V_4_ through V_6_, there was progressive amplification of the late R′-wave and T-wave. Taken together, these changes show that the model depends most on R-wave enhancement and S-wave narrowing—especially in the lateral leads—when deciding that an LBBB tracing indicates LVSD.

In parallel, we performed univariate correlation analyses between conventional ECG parameters and AI-ECG–predicted LVSD probabilities in the external validation cohort (Figure 4B). Consistent with the GCX findings, prolonged QRS duration and QTc interval were strongly associated with higher predicted probabilities, highlighting their relevance in the model's decision-making process.

## Discussion

This study is the first study to address model development strategies for predicting LVSD using AI-ECG in LBBB patients. In this study, we demonstrated 3 key findings. First, the transfer learning–based model (Model 4) achieved the highest discrimination for detecting LVSD in LBBB patients, although the general model (Model 1) performed comparably, underscoring the viability of a single, broadly trained AI-ECG tool. Second, our results reveal that the composition of the training data set influences model performance in challenging subgroups like LBBB, and we validated these findings using a well-curated external cohort. Third, the AI-ECG shows promise as a digital biomarker not only for diagnosing current LVSD but also for predicting future LVSD.

### Model development strategy and transfer learning

Despite the extensive body of AI-ECG research, few studies discuss key model development strategies such as data labeling and selection of development cohorts.^16^ Our work addresses this gap by exploring how these factors impact diagnostic performance. Our findings underscore the critical role of model development strategy—specifically, how the training set is constructed—in enhancing diagnostic performance. We observed that fine-tuning a general AI-ECG model on LBBB-specific data resulted in a modest improvement in AUROC compared to the general model alone. This suggests that incorporating pathophysiology-specific nuances during training can boost the detection of LVSD in challenging subgroups, as previous studies have shown that models trained on broad data sets tend to underperform in LBBB patients but can be improved through transfer learning.^25^^,^^26^ Our results are consistent with prior research, which indicates that fine-tuning a pretrained model on a limited, high-risk cohort can significantly outperform models developed solely on restricted data.^27^ These findings highlight that leveraging knowledge from large, diverse data sets to inform specialized contexts can improve accuracy when scarce data or phenotypes are distinct. Nevertheless, the incremental benefits of transfer learning must be balanced against its increased complexity and resource demands. In other words, for widespread screening applications, a robust general model—provided it is trained on a sufficiently diverse data set—may be more practical, with transfer learning reserved for situations where the general model's performance is inadequate.

While the AUROC reflects overall discriminative ability, in real-world screening settings, the degree to which specificity improves when sensitivity is fixed at a clinically relevant level (eg, 90%) is a key determinant of the burden of unnecessary follow-up testing.^23^^,^^28^ In our analysis, Models 1 (general AI-ECG, AiTiALVSD) and 4 (transfer learning–enhanced LBBB) achieved specificities of 0.702 and 0.718, respectively, at 90% sensitivity, indicating only a modest performance gap between the approaches. This suggests that the general model may offer screening utility comparable to that of the transfer learning model. However, by achieving a 1.6 percentage-point higher specificity than Model 1, Model 4 could reduce the number of false positives by approximately 16 per 1,000 individuals screened, thereby potentially alleviating patient anxiety and conserving medical resources. In contrast, Models 2 and 3 exhibited specificities of only 0.596 and 0.542, respectively, risking excessive false positives in pursuit of sensitivity. These findings highlight that even a small gain in specificity at a fixed sensitivity can meaningfully reduce false positives and related costs, so threshold-specific metrics should complement AUROC when selecting screening models.

### Clinical applications and digital biomarker potential

From a clinical perspective, our findings imply 2 key points. First, AI-enabled ECG can be an effective screening tool for occult LVSD, even in patients with LBBB. Although LBBB complicates traditional ECG interpretation—often necessitating echocardiography—our general model reliably extracts predictive features for LVSD. This suggests that, in a real-world setting, an AI-ECG could broadly screen patients and flag high-risk LBBB cases for confirmatory imaging, thereby enabling earlier intervention in heart failure management.

Second, our work reinforces the concept of AI-ECG outputs as digital biomarkers with prognostic significance beyond mere diagnosis.^29^ A low EF prediction from the AI-ECG identifies current LVSD and correlates with an increased risk of future adverse outcomes. In the context of LBBB—where early signs of progressive myocardial dysfunction may be subtle—using AI-ECG as a digital biomarker could help differentiate benign conduction variants from early pathological changes. Overall, the dual role of AI-ECG in screening and risk stratification may offer a paradigm shift in preventive cardiology by providing a timely, noninvasive method for early detection and intervention.

In summary, the exceptionally high negative predictive performance demonstrated by AI-ECG in our study means that a negative result can reliably exclude LVSD, allowing clinicians to defer echocardiography in these patients, conserve resources, and reduce patient burden. We emphasize this high negative predictive power and its resource-saving potential in LBBB patients, and we propose that integrating simple clinical variables or optimizing operating thresholds could further improve specificity and enhance positive predictive yield in this high-risk subgroup.

In our false-positive LBBB cohort (n = 366), the median LV EF was 55% (IQR: 47%-60%). Notably, 20.5% had undergone valvular replacement surgery, 11.2% had prior percutaneous coronary intervention or coronary artery bypass grafting, 2.0% had ventricular septal myectomy, and 6.3% had permanent pacemaker implantation. These comorbid structural heart diseases likely induce ECG changes that mimic LVSD despite preserved EF, explaining many false positive AI-ECG classifications.

### Subgroup performance, bias, and explainability

Our subgroup analyses revealed that the general model (Model 1) performed better in younger patients, those without IHD, and those with DM, while its accuracy was lower in older patients and those with IHD. This pattern is consistent with prior studies suggesting that younger individuals exhibit a stronger association between abnormal ECG findings and clinically significant pathology, whereas older patients more frequently present nonspecific ECG abnormalities. These age-related differences likely stem from both biological variability in disease presentation and data imbalance in the training set, as supported by previous literature.^25^^,^^30^ This discrepancy likely reflects an overrepresentation of younger and diabetic patients in the training data set, causing the model to learn decision boundaries tuned to these demographics. Consequently, when applied to less-represented subgroups—such as older individuals or patients with IHD—the model's performance diminished, indicating a form of data-induced bias. These findings underscore broader concerns about bias and fairness in AI models, where uneven performance across subpopulations could exacerbate health disparities. To ensure uniform accuracy, the training data set must be as diverse and representative as the intended patient population. In the interim, clinicians should exercise caution, particularly when interpreting AI-ECG results in under-represented groups. Addressing these biases through methods like data set rebalancing, data augmentation, or fairness-oriented algorithms is essential for the equitable deployment of AI-ECG tools.^31^

Given the performance nuances and potential biases, explainable AI (XAI) is critical, especially in LBBB where altered ECG waveforms challenge ventricular function interpretation. To address this, we employed XAI techniques—including a novel generative counterfactual explanation framework—to clarify our model's decisions.^24^ Unlike traditional saliency maps that merely highlight regions, GCX generates modified ECG waveforms to show how minimal changes can alter predictions, offering intuitive and clinically relevant insights. Our GCX findings revealed that as the AI-ECG score increased, the ECG waveform exhibited progressive prolongation of QRS duration and QTc interval, along with subtle changes in T-wave morphology. These patterns suggest that the model relies heavily on temporal and morphological features associated with electrical dyssynchrony to predict LVSD in LBBB patients. Although our GCX framework offers physiologically plausible interpretations, its clinical usefulness has not yet been formally evaluated. Future work should include prospective reader studies or expert validation to determine whether GCX outputs are intuitively understandable and actionable by cardiologists in real-world settings. Tracing these counterfactual waveform trajectories also allowed us to approximate the electrophysiologic alterations—such as regional conduction delays and repolarization shifts—that underlie EF reduction in dyssynchronous ventricles, providing a noninvasive window into mechanical dysfunction. Furthermore, incorporating XAI enhances transparency and builds trust, ensuring that clinicians can understand and interrogate AI outputs.

### Study Limitations

This study has several limitations. First, our analysis is retrospective, relying on historical records and a moderate-sized external validation cohort drawn from a similar healthcare context. Consequently, the real-time performance of our AI-ECG model and its generalizability to other populations remain uncertain. Second, we focused exclusively on LBBB patients with LVSD defined by a specific EF threshold, which may limit the applicability of our findings to other conduction abnormalities or cardiac conditions. Third, although our transfer learning approach yielded an improvement in AUROC, the absolute gain was modest, and we did not evaluate whether this incremental benefit translates into meaningful clinical outcomes. Moreover, implementing transfer learning adds complexity by requiring prior identification of LBBB for appropriate model routing, and we did not explore a unified model capable of adapting internally to such features. Fourth, our use of explainability techniques such as the GCX framework was largely qualitative, and we did not formally assess how well these explanations align with expert reasoning or impact clinical decision-making.

These limitations suggest several areas for future research. First, we need prospective clinical trials to test our model in real-world settings and to compare the performance of using various models. Second, exploring advanced modeling techniques—such as multi-task learning, domain adaptation, or meta-learning—might help our model better adjust to changes in the data. Finally, it is important to include more diverse data sets and apply fairness techniques. We also need to quantitatively evaluate XAI methods to understand their impact on clinicians' trust.

## Conclusions

LVSD in LBBB patients poses a diagnostic challenge due to unique ECG alterations and clinical heterogeneity. Our study demonstrates that a broadly trained AI-ECG model reliably detects LVSD and is equivalent or superior to models using LBBB-specific data sets. Transfer learning yields modest performance improvements. Nonetheless, traditional quantitative metrics may not fully reflect clinical utility. Comprehensive external and prospective evaluations in representative clinical populations are essential to confirm these findings and guide the integration of AI-ECG screening into routine practice.

Perspectives **COMPETENCY IN MEDICAL KNOWLEDGE:** Artificial intelligence–enabled ECG models can detect LVSD in patients with LBBB, a subgroup traditionally considered challenging for ECG-based screening. This study demonstrates that model development strategies, particularly training data set composition and transfer learning approaches, significantly influence diagnostic performance. Clinicians and developers should carefully consider cohort selection and model adaptation when applying AI-ECG tools to heterogeneous or high-risk populations like LBBB.

**TRANSLATIONAL OUTLOOK 1:** Transfer learning using general model weights fine-tuned on LBBB-specific data yielded improved diagnostic accuracy. However, the general model also showed comparable performance, suggesting that well-developed general models can still be effective and serve as a foundation for further refinement in rare or complex subgroups.

**TRANSLATIONAL OUTLOOK 2:** Prospective validation across diverse clinical settings and streamlined XAI integration are needed to support clinical adoption and trust.

## Funding support and author disclosures

This research was supported by a grant of the Korea Health Technology R&D Project through the Korea Health Industry Development Institute (KHIDI), funded by the Ministry of Health & Welfare, Republic of Korea (RS-2023-KH134607). Drs H.S. Lee, Kang, Han, Yoo, Jang, Jo, Son, M.S. Lee, and Kwon are employees of Medical AI Co, Ltd and hold stocks in the company. All other authors have reported that they have no relationships relevant to the contents of this paper to disclose.

## References

[1] Imanishi R., Seto S., Ichimaru S., Nakashima E., Yano K., Akahoshi M. (2006). Prognostic significance of incident complete left bundle branch block observed over a 40-year period. Am J Cardiol.

[2] Eriksson P., Hansson P.O., Eriksson H., Dellborg M. (1998). Bundle-branch block in a general male population: the study of men born 1913. Circulation.

[3] Kowlgi G.N., Tan A.Y., Kaszala K. (2022). Left ventricular dyssynchrony as marker of early dysfunction in premature ventricular contraction-induced cardiomyopathy. Front Cardiovasc Med.

[4] Huizar J.F., Tan A.Y., Kaszala K., Ellenbogen K.A. (2021). Clinical and translational insights on premature ventricular contractions and PVC-induced cardiomyopathy. Prog Cardiovasc Dis.

[5] Hamdy R.M., Osama H., Fereig H.M. (2022). Evaluation of cardiac mechanical dyssynchrony in heart failure patients using current echo-doppler modalities. J Cardiovasc Imaging.

[6] Sharma S., Barot H.V., Schwartzman A.D. (2020). Risk and predictors of dyssynchrony cardiomyopathy in left bundle branch block with preserved left ventricular ejection fraction. Clin Cardiol.

[7] Lee S.J., McCulloch C., Mangat I., Foster E., De Marco T., Saxon L.A. (2003). Isolated bundle branch block and left ventricular dysfunction. J Card Fail.

[8] Elias P., Jain S.S., Poterucha T. (2024). Artificial intelligence for cardiovascular care-part 1: advances: JACC Review Topic of the Week. J Am Coll Cardiol.

[9] Yao X., Rushlow D.R., Inselman J.W. (2021). Artificial intelligence-enabled electrocardiograms for identification of patients with low ejection fraction: a pragmatic, randomized clinical trial. Nat Med.

[10] Bachtiger P., Petri C.F., Scott F.E. (2022). Point-of-care screening for heart failure with reduced ejection fraction using artificial intelligence during ECG-enabled stethoscope examination in London, UK: a prospective, observational, multicentre study. Lancet Digit Health.

[11] Kwon J.M., Kim K.H., Jeon K.H. (2019). Development and validation of deep-learning algorithm for electrocardiography-based heart failure identification. Korean Circ J.

[12] Adedinsewo D.A., Morales-Lara A.C., Afolabi B.B. (2024). Artificial intelligence guided screening for cardiomyopathies in an obstetric population: a pragmatic randomized clinical trial. Nat Med.

[13] Tomoaia R., Harrison P., Bevis L. (2024). CMR characterization of patients with heart failure and left bundle branch block. Eur Heart J Imaging Methods Pract.

[14] Vokinger K.N., Feuerriegel S., Kesselheim A.S. (2021). Mitigating bias in machine learning for medicine. Commun Med (Lond).

[15] Gianfrancesco M.A., Tamang S., Yazdany J., Schmajuk G. (2018). Potential biases in machine learning algorithms using electronic health record data. JAMA Intern Med.

[16] Vrudhula A., Stern L., Cheng P.C. (2024). Impact of case and control selection on training artificial intelligence screening of cardiac amyloidosis. JACC Adv.

[17] Heidenreich P.A., Bozkurt B., Aguilar D. (2022). 2022 AHA/ACC/HFSA guideline for the management of heart failure: executive summary: a report of the American College of Cardiology/American Heart Association Joint Committee on clinical practice guidelines. J Am Coll Cardiol.

[18] McDonagh T.A., Metra M., Adamo M. (2022). 2021 ESC guidelines for the diagnosis and treatment of acute and chronic heart failure: developed by the Task Force for the diagnosis and treatment of acute and chronic heart failure of the European Society of Cardiology (ESC) with the special contribution of the Heart Failure Association (HFA) of the ESC. Rev Esp Cardiol (Engl Ed).

[19] Jung Y.M., Kang S., Son J.M. (2023). Electrocardiogram-based deep learning model to screen peripartum cardiomyopathy. Am J Obstet Gynecol MFM.

[20] Jeong J.H., Kang S., Lee H.S. (2024). Deep learning algorithm for predicting left ventricular systolic dysfunction in atrial fibrillation with rapid ventricular response. Eur Heart J Digit Health.

[21] Quer G., Arnaout R., Henne M., Arnaout R. (2021). Machine learning and the future of cardiovascular care: JACC State-of-the-Art review. J Am Coll Cardiol.

[22] Tadesse G.A., Zhu T., Liu Y. (2019). Cardiovascular disease diagnosis using cross-domain transfer learning. Annu Int Conf IEEE Eng Med Biol Soc.

[23] Ulloa-Cerna A.E., Jing L., Pfeifer J.M. (2022). rECHOmmend: an ECG-based machine learning approach for identifying patients at increased risk of undiagnosed structural heart disease detectable by echocardiography. Circulation.

[24] Jang J.-H., Jo Y.-Y., Kang S. (2025). A novel XAI framework for explainable AI-ECG using generative counterfactual XAI (GCX). Sci Rep.

[25] Harmon D.M., Carter R.E., Cohen-Shelly M. (2022). Real-world performance, long-term efficacy, and absence of bias in the artificial intelligence enhanced electrocardiogram to detect left ventricular systolic dysfunction. Eur Heart J Digit Health.

[26] König S., Hohenstein S., Nitsche A. (2023). Artificial intelligence-based identification of left ventricular systolic dysfunction from 12-lead electrocardiograms: external validation and advanced application of an existing model. Eur Heart J Digit Health.

[27] Vaid A., Jiang J.J., Sawant A. (2022). Automated determination of left ventricular function using electrocardiogram data in patients on maintenance hemodialysis. Clin J Am Soc Nephrol.

[28] Maxim L.D., Niebo R., Utell M.J. (2014). Screening tests: a review with examples. Inhal Toxicol.

[29] Al-Alusi M.A., Friedman S.F., Kany S. (2025). A deep learning digital biomarker to detect hypertension and stratify cardiovascular risk from the electrocardiogram. NPJ Digit Med.

[30] Elias P., Poterucha T.J., Rajaram V. (2022). Deep learning electrocardiographic analysis for detection of left-sided valvular heart disease. J Am Coll Cardiol.

[31] Noseworthy P.A., Attia Z.I., Brewer L.C. (2020). Assessing and mitigating bias in medical artificial intelligence: the effects of race and ethnicity on a deep learning model for ECG analysis. Circ Arrhythm Electrophysiol.

