# Supplementary material for: Artificial Intelligence–Enabled ECG Screening for LVSD in LBBB: Evaluating Model Development and Transfer Learning Approaches
Source: JACC Adv. 2025 Aug 21;4(9):102089. doi: 10.1016/j.jacadv.2025.102089 (PMC12398843; doi:10.1016/j.jacadv.2025.102089)
Supplement: Supplementary data [file mmc1.pdf]

TRIPOD-AI Checklist for: Artificial Intelligence-Enabled ECG Screening for LVSD in LBBB

This document provides the completed TRIPOD-AI checklist for the submitted manuscript. Each item is addressed according to its location in the manuscript.

Section/Topic	Item No.	Checklist Item	Reported on Page/Section
Title and Abstract	1	Identify the study as developing or evaluating the performance of a multivariable prediction model, the target population, and the outcome to be predicted	Title Page
Title and Abstract	2	Structured summary of objectives, design, setting, participants, sample size, predictors, outcome, analysis, results, and conclusions	Abstract (Page 2)
Introduction	3a–c	Rationale, context, inequalities	Introduction (Page 3)
Introduction	4	Specify whether the study describes model development, validation, or both	Introduction (Page 4)
Methods	5a–b	Data source and dates	Methods (Page 5)
Methods	6a–c	Setting, eligibility, treatments	Methods (Pages 5–6)
Methods	7	Preprocessing and quality checking	Methods (Page 6)
Methods	8a–c	Outcome definition and assessment	Methods (Page 6)
Methods	9a–c	Input variables, measurement	Methods (Page 6) and Supplementary Methods 1
Methods	10	Study size and justification	Methods (Page 6), Introduction (Page 4)
Methods	11	Handling missing data	Methods (Page 6)
Methods	12a–g	Model type, training, validation, performance metrics	Methods (Pages 6–8), Supplementary Methods 2
Methods	13	Handling of imbalance	Supplementary Methods 2
Methods	14	Fairness strategies and rationale	Discussion (Page 13)
Methods	15	Output type and thresholding	Methods (Page 7), Supplementary Methods 1

Methods	16	Differences in cohorts	Methods (Page 6), Results (Page 9)
Methods	17	IRB approval and waiver of consent	Methods (Page 5)
Open Science	18a	Funding source and role	Funding (Page 2)
Open Science	18b	Conflicts and disclosures	Page 2
Open Science	18c	Protocol availability	Not prepared (stated on Page 2)
Open Science	18d	Trial registration	Not registered (Page 2)
Open Science	18e	Availability of data	Discussion (Page 13)
Open Science	18f	Availability of code	Methods (Page 7), Discussion (Page 13)
Patient & Public Involvement	19	Involvement during study	Not involved (Page 2)
Results	20a	Flow of participants	Figure 1 (Page 8)
Results	20b	Characteristics, missing data	Table 1 (Page 9), Supplementary Table 1
Results	20c	Predictor distributions across cohorts	Supplementary Figure 1
Results	21	N and events per model	Methods (Page 6), Table 2 (Page 10)
Results	22	Model formula, implementation	Supplementary Methods 2
Results	23a–b	Performance with CI, subgroup	Tables 2 and 3 (Page 10–11), Figure 3
Results	24	Recalibration or model updating	Not applicable
Discussion	25	Interpretation and fairness	Discussion (Pages 12–14)
Discussion	26	Limitations and implications	Discussion (Pages 14–15)
Discussion	27a–c	Handling input quality, user interaction, next steps	Discussion (Pages 13–15)

Prepared in accordance with TRIPOD-AI guidelines.

Supplemental Methods 1. AI-ECG Score Computation and Thresholding

Score Definition

All four AI-ECG models generate a continuous probability score indicating the likelihood of left ventricular systolic dysfunction (LVSD). The neural network produces a logit value in its final layer, which is passed through a sigmoid activation function to generate a probability ranging from 0 to 1. This value is then scaled to a 0–100 range and reported with one decimal precision. The LVSD probability score represents the model's confidence that the given ECG corresponds to a patient with LVSD.

Calibration

To enhance the reliability of the predicted probability scores, temperature scaling was applied as a post-hoc calibration method. The temperature parameter was optimized on a held-out validation subset of the training data by minimizing the negative log-likelihood. This procedure adjusts the sharpness of the probability distribution without affecting the underlying model predictions, ensuring better alignment between predicted probabilities and actual event likelihoods.

Score Distribution

The distribution of AI-ECG scores was analyzed across the training, validation, and external test datasets. As expected, the distributions demonstrated greater separation between LVSD and non-LVSD cases in the training and validation sets, with some overlap in the external test set consistent with real-world diagnostic ambiguity. Representative histograms of score distributions are shown in Supplementary Figure 1.

Threshold Selection (Binary Classification Cut-off)

For binary classification tasks, a single operating threshold was selected using the Youden Index (sensitivity + specificity – 1) on the internal validation set derived from the training

cohort. This threshold maximizes balanced classification performance. Once established, this cut-off was fixed and applied consistently across all evaluation datasets, including internal test and external validation sets, to ensure fair and reproducible model comparison while preventing information leakage.

Supplemental Methods 2. Fine-Tuning Protocol for Model 4

Layer Freezing Strategy

We adopted a full fine-tuning strategy for Model 4. All layers, including the pre-trained backbone network (Model 1: AiTiALVSD), were unfrozen and updated during training. No layers were frozen.

Training Hyperparameters

- Epochs: 5
- Batch size: 128
- Optimizer: Adam
- Learning rate: 1.07×10^{-4}
- Weight decay: 2.88×10^{-6}
- Learning rate scheduler: CosineWarmup

  The learning rate and weight decay were selected based on hyperparameter tuning using validation set performance.

Loss Function

We employed Focal Loss to address class imbalance in the binary LVSD classification task.

Model Architecture

The backbone architecture was a ResNet-based convolutional neural network tailored for 12-lead ECG signals. It consisted of three convolutional layers, each containing four residual blocks.

Supplemental Figure 1: Histogram of AI-ECG score distributions across training, validation, and test sets

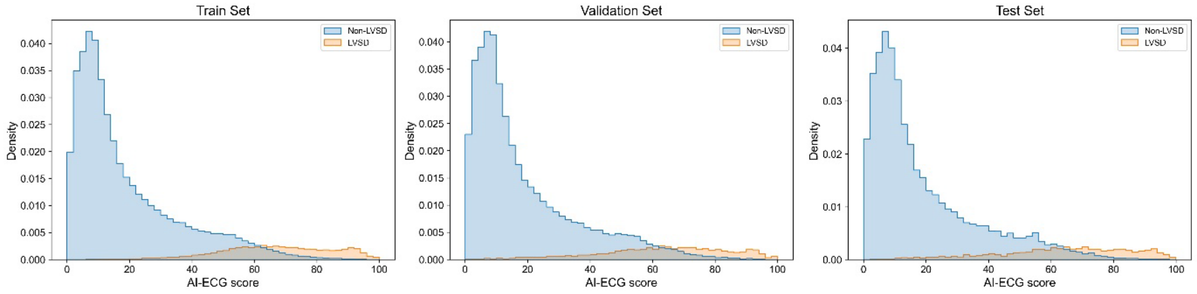

This figure presents the distribution of AI-ECG scores for patients with and without LVSD in the training, validation, and external test sets. AI-ECG scores are plotted as density curves ranging from 0 to 100, representing the model's predicted probability of LVSD. In all datasets, non-LVSD cases (blue) are concentrated at lower scores, while LVSD cases (orange) demonstrate a broader and right-shifted distribution, reflecting the model's discriminative capability.

AI-ECG, artificial intelligence–enabled electrocardiogram; LVSD, left ventricular systolic dysfunction.

Supplemental Table 1. Baseline characteristics of internal validation set according to LVSD

	LBBB			
	Total (n=4355)	LVSD (n=1796)	non-LVSD (n=2559)	p-value
Age	72.2 ± 11.3	72.0 ± 11.1	72.4 ± 11.5	0.303
Sex (male)	1876 (43.1)	958 (53.3)	918 (35.9)	<0.001
Height	158.1 ± 10.2	159.9 ± 9.4	156.9 ± 10.5	<0.001
Weight	60.9 ± 12.5	60.2 ± 11.7	61.3 ± 13.0	0.003
BMI	24.8 ± 17.1	23.5 ± 5.3	25.7 ± 21.6	<0.001
**Past medical illness**				
Diabetes mellitus	1075 (24.7)	564 (31.4)	511 (20.0)	<0.001
Hypertension	2353 (54.0)	931 (51.8)	1422 (55.6)	0.206
Ischemic heart disease	1972 (45.3)	906 (50.4)	1066 (41.7)	<0.001
Heart failure	2732 (62.7)	1524 (84.9)	1208 (47.2)	<0.001
Atrial fibrillation	1183 (27.2)	534 (29.7)	649 (25.4)	0.037
Chronic kidney disease	431 (9.9)	230 (12.8)	201 (7.9)	<0.001
Stroke	471 (10.8)	185 (10.3)	286 (11.2)	0.933
**Electrocardiogram**				
HR, bpm	75.9 ± 18.1	78.7 ± 19.5	73.9 ± 16.8	<0.001
PR interval, ms	188.5 ± 43.5	187.0 ± 42.9	189.5 ± 43.9	0.086
QT interval, ms	456.6 ± 52.9	454.0 ± 53.3	458.5 ± 52.5	0.006
QRS duration, ms	152.2 ± 17.9	156.1 ± 20.1	149.5 ± 15.5	<0.001
QTc interal, ms	504.6 ± 40.5	510.5 ± 41.9	500.5 ± 38.9	<0.001
P axis	43.1 ± 42.3	45.0 ± 41.0	41.9 ± 43.2	0.042
R axis	-8.7 ± 34.8	-11.3 ± 36.9	-6.9 ± 33.1	<0.001
T axis	134.2 ± 61.1	141.5 ± 67.1	129.1 ± 55.8	<0.001
**LVEF**	43.3 ± 14.0	28.5 ± 7.2	53.8 ± 5.8	<0.001

Values are expressed as n (%), mean ± standard deviation.

LBBB, left bundle branch block; LVSD, left ventricular systolic dysfunction; BMI, body mass index; HR, heart rate; LVEF, left ventricular ejection fraction

Supplemental Table 2. Subgroup Analysis of the General AI-ECG Model

	AUROC<br>(95% CI)	AUPRC<br>(95% CI)	Sensitivity<br>(95% CI)	Specificity<br>(95% CI)	PPV<br>(95% CI)	NPV<br>(95% CI)
**Age ≥ 65<br>(n=960)**	0.884<br>(0.863–0.904)	0.820<br>(0.801–0.839)	0.957<br>(0.940–0.974)	0.423<br>(0.377–0.471)	0.659<br>(0.624–0.695)	0.895<br>(0.853–0.934)
**Age < 65<br>(n=374)**	0.940<br>(0.918–0.960)	0.814<br>(0.786–0.843)	0.990<br>(0.974–1.000)	0.386<br>(0.315–0.462)	0.633<br>(0.577–0.689)	0.972<br>(0.928–1.000)
**Male<br>(n=571)**	0.905<br>(0.880–0.929)	0.852<br>(0.830–0.873)	0.980<br>(0.965–0.994)	0.355<br>(0.296–0.422)	0.712<br>(0.669–0.751)	0.917<br>(0.857–0.974)
**Female<br>(n=763)**	0.890<br>(0.865–0.914)	0.787<br>(0.764–0.810)	0.952<br>(0.928–0.973)	0.444<br>(0.397–0.490)	0.600<br>(0.561–0.639)	0.914<br>(0.874–0.952)
**HTN<br>(n=644)**	0.897<br>(0.872–0.921)	0.807<br>(0.783–0.830)	0.945<br>(0.920–0.969)	0.445<br>(0.391–0.503)	0.640<br>(0.598–0.681)	0.886<br>(0.833–0.937)
**Non-HTN<br>(n=690)**	0.900<br>(0.878–0.922)	0.827<br>(0.808–0.849)	0.984<br>(0.970–0.995)	0.381<br>(0.326–0.438)	0.662<br>(0.623–0.703)	0.950<br>(0.913–0.984)
**DM<br>(n=357)**	0.939<br>(0.911–0.963)	0.860<br>(0.835–0.885)	0.991<br>(0.977–1.000)	0.393<br>(0.314–0.474)	0.724<br>(0.673–0.774)	0.964<br>(0.913–1.000)
**Non-DM<br>(n=977)**	0.881<br>(0.859–0.901)	0.801<br>(0.782–0.819)	0.955<br>(0.935–0.973)	0.420<br>(0.376–0.462)	0.624<br>(0.591–0.658)	0.903<br>(0.863–0.940)
**IHD<br>(n=627)**	0.873<br>(0.845–0.899)	0.820<br>(0.796–0.842)	0.947<br>(0.923–0.968)	0.423<br>(0.365–0.479)	0.663<br>(0.620–0.704)	0.869<br>(0.814–0.921)
**non-IHD<br>(n=707)**	0.922<br>(0.901–0.939)	0.816<br>(0.795–0.837)	0.984<br>(0.969–0.995)	0.403<br>(0.354–0.454)	0.641<br>(0.600–0.681)	0.958<br>(0.920–0.987)
**HF<br>(n=913)**	0.895<br>(0.874–0.915)	0.858<br>(0.842–0.876)	0.971<br>(0.956–0.983)	0.389<br>(0.339–0.441)	0.727<br>(0.697–0.760)	0.887<br>(0.834–0.935)
**non-HF<br>(n=421)**	0.880<br>(0.843–0.916)	0.707<br>(0.674–0.740)	0.949<br>(0.912–0.984)	0.444<br>(0.382–0.502)	0.450<br>(0.392–0.507)	0.948<br>(0.908–0.984)
	0.888	0.869	0.991	0.292	0.741	0.939

**AF (n=323)**	(0.848–0.924)	(0.845–0.895)	(0.977–1.000)	(0.207–0.385)	(0.692–0.792)	(0.846–1.000)
**non-AF (n=1011)**	0.897 (0.876–0.917)	0.798 (0.777–0.816)	0.956 (0.937–0.973)	0.438 (0.395–0.483)	0.618 (0.581–0.652)	0.913 (0.876–0.946)

AUROC, area under the receiver operating characteristic curve; AUPRC, area under the precision-recall curve; PPV, positive predictive value; NPV, negative predictive value; CI, confidence interval; HTN, hypertension; DM, diabetes mellitus; IHD, ischemic heart disase; HF, heart failure; AF, atrial fibrillation
